# Supplementary material for: Dioscorea nipponica Makino Rhizome Extract and Its Active Compound Dioscin Protect against Neuroinflammation and Scopolamine-Induced Memory Deficits
Source: Int J Mol Sci. 2022 Sep 1;23(17):9923. doi: 10.3390/ijms23179923 (PMC9456145; doi:10.3390/ijms23179923)

Dioscorea nipponica Makino rhizome extract and its active compound dioscin protect against neuroinflammation and scopolamine-induced memory deficits

Shofiul Azam [1#], Yon-Suk Kim [2#], Md Jakaria [1,4], Ye-Ji Yu [1], Jaeyong Ahn [1], In-Su Kim [1,3], Dong-Kug Choi [1,3*]

[1]Department of Applied Life Sciences, Graduate School, BK21 Program, Konkuk University, Chungju 27478, South Korea

[2]BKplus GLOCAL education program of nutraceuticals development, Konkuk University, Chungju 27478, South Korea

[3]Department of Biotechnology, College of Biomedical and Health Science, and Research Institute of Inflammatory Disease (RID), Konkuk University, Chungju 27478, South Korea

[4]Melbourne Dementia Research Centre, The Florey Institute of Neuroscience and Mental Health, The University of Melbourne, Parkville, VIC 3052, Australia

[#]Both authors have contributed equally

*Correspondence: choidk@kku.ac.kr; Tel.: +82-43-840-3610; Fax: +82-43-840-3872

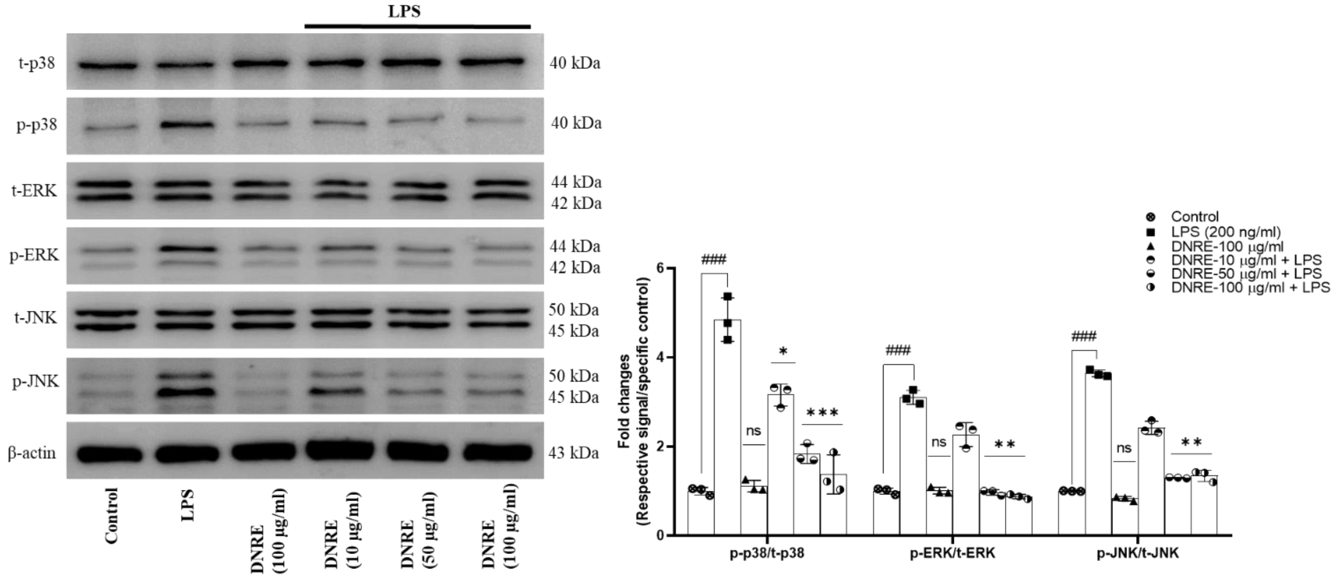

Supplementary Figure S1: DNRE effects on MAPK signalling. Protein expression of mediators of MAPK signalling in LPS treated BV-2 cells with or without DNRE and densitometric calculation using ImageJ. ns-not significant compared with non-treated; ##p<0.01, ###p<0.001 compared with non-treated; **p<0.01, ***p<0.001 compared LPS vs LPS+DNRE.

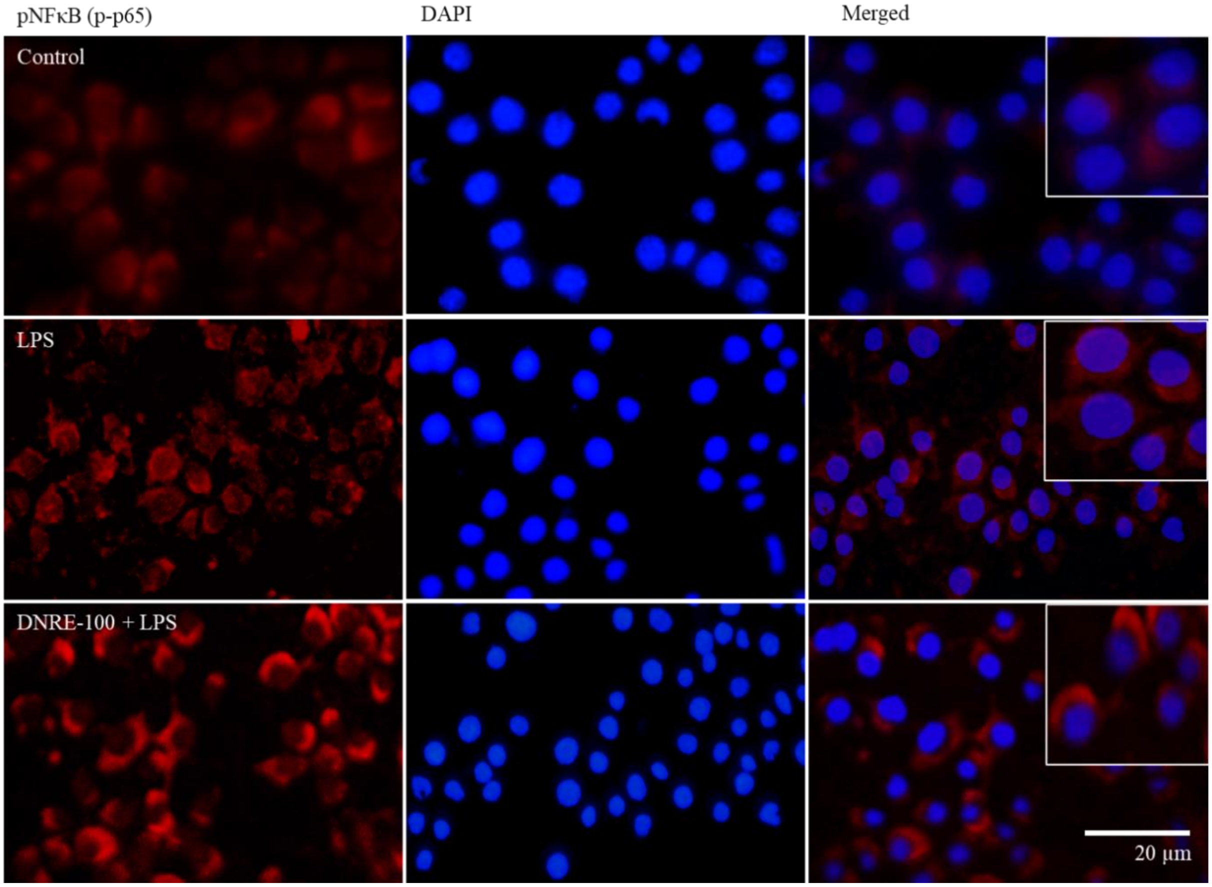

Supplementary Figure S2: BV-2 cells were treated with or without LPS and DNRE for 24 h. Cells were stained with anti-phosphorylated NFκB (p-p65). Images were taken using Nikon fluorescence imaging system (scale bar = 20 µm).

Full unedited blots sample for respective figures-

Fig 3

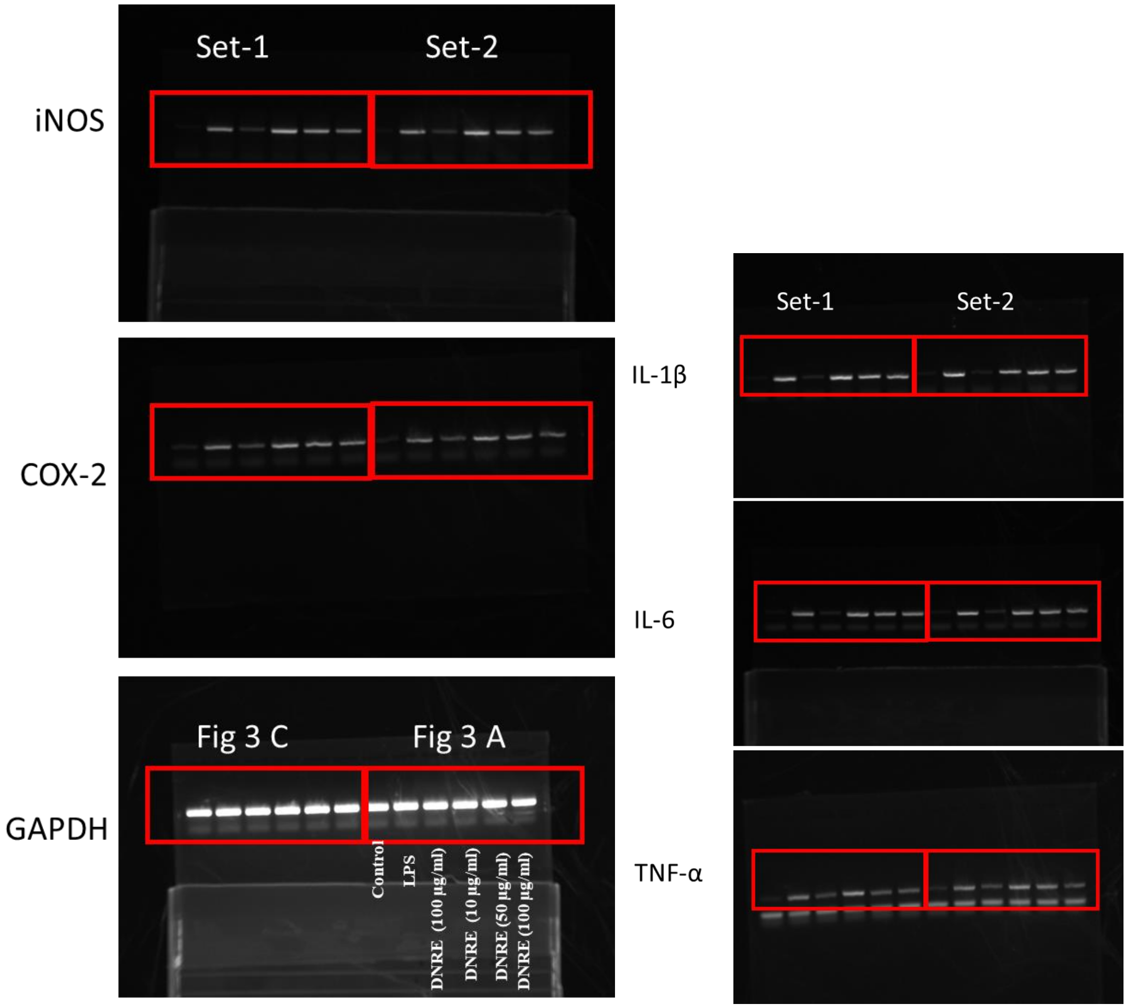

Fig 3 B

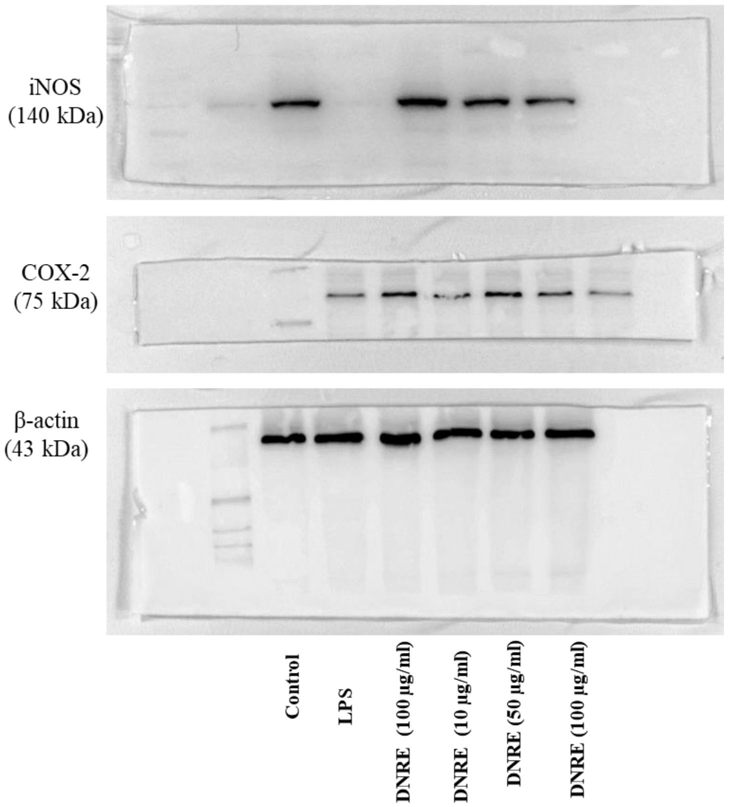

Fig 4 A

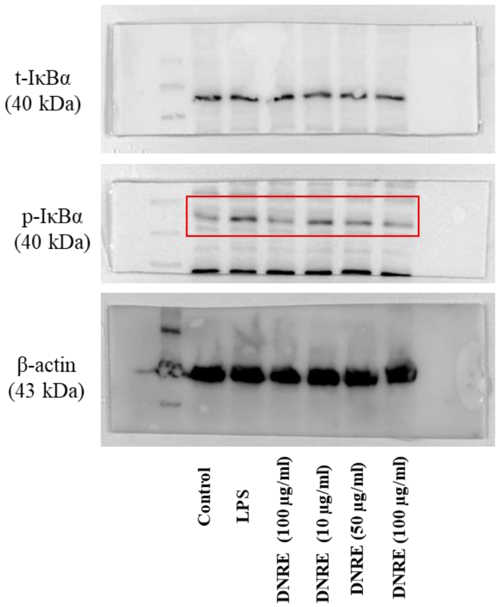

Fig 4 B

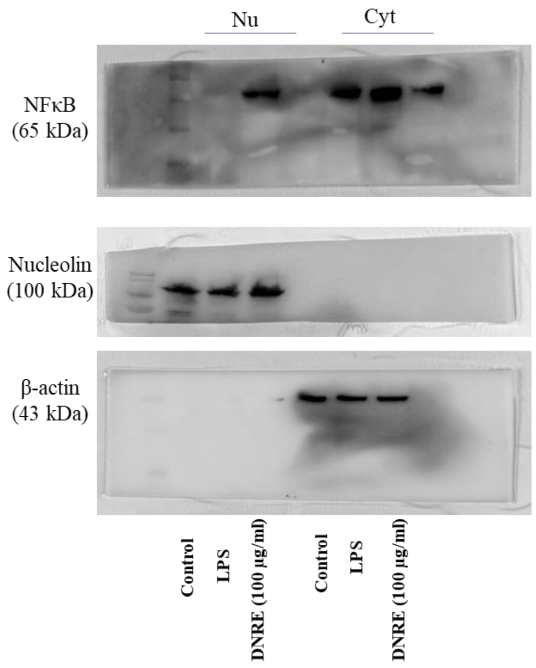

Fig 5 B

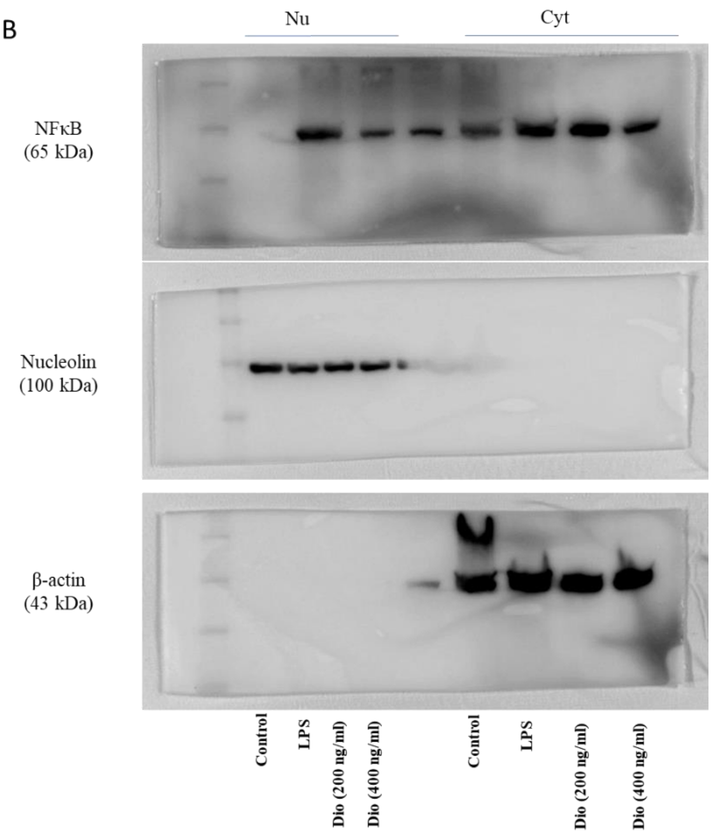

Fig 7 A

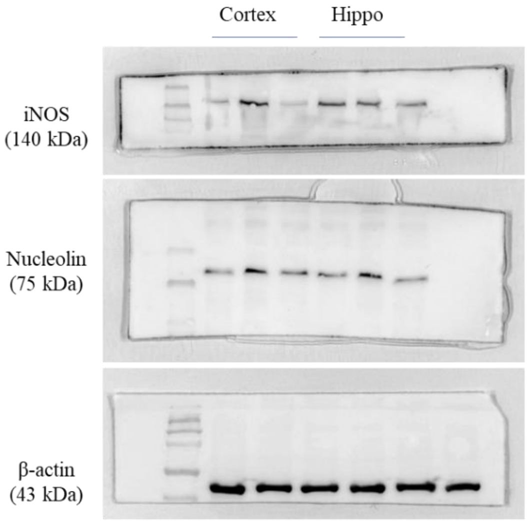

Fig 7 B

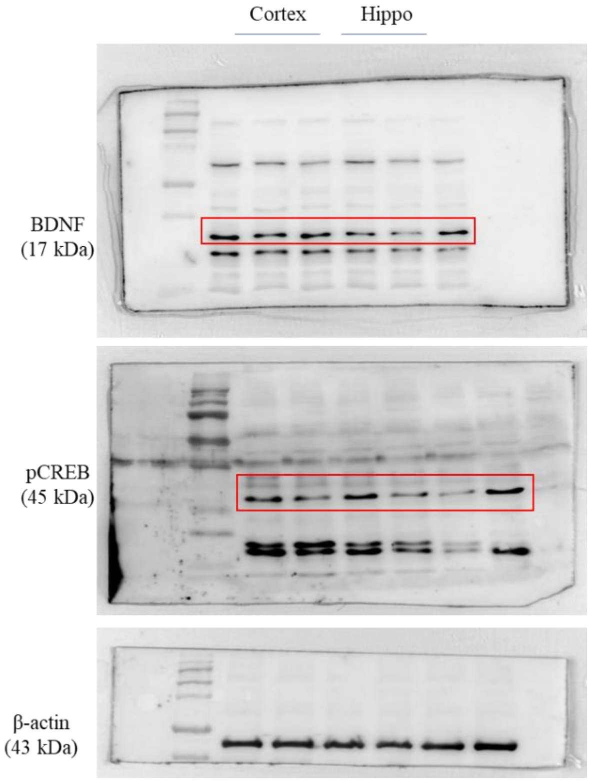

Supplementary Figure S1

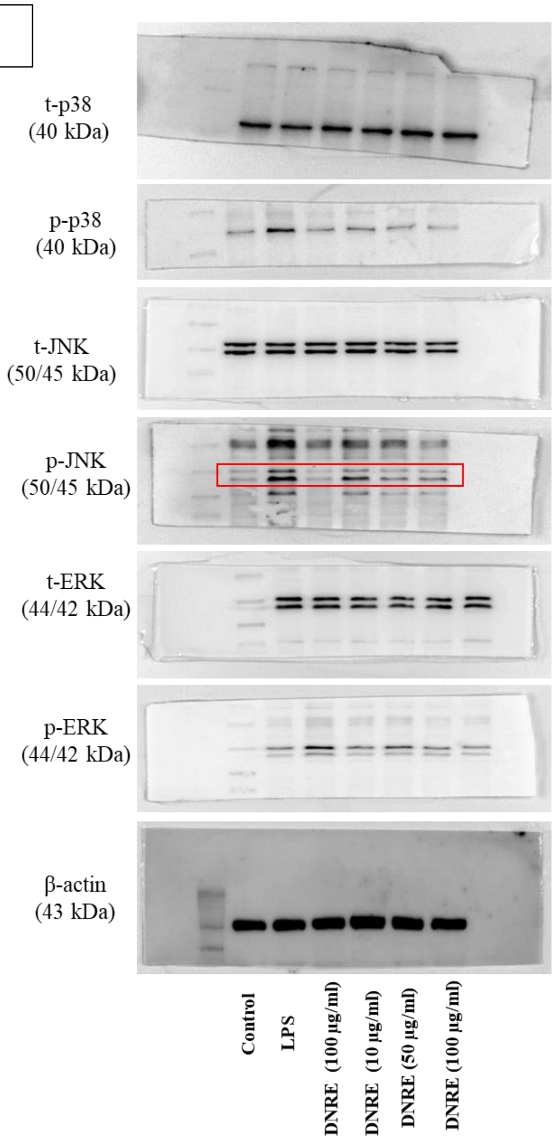

Supplement: Supplementary file 1 [file ijms-23-09923-s001.zip › ijms-1823621-supplementary.PDF]
